# Complex relationships between climate and reproduction in a resident montane bird

**DOI:** 10.1098/rsos.230554

**Published:** 2023-06-21

**Authors:** Lauren E. Whitenack, Joseph F. Welklin, Carrie L. Branch, Benjamin R. Sonnenberg, Angela M. Pitera, Dovid Y. Kozlovsky, Lauren M. Benedict, Virginia K. Heinen, Vladimir V. Pravosudov

**Affiliations:** ^1^ Department of Biology, Ecology, Evolution and Conservation Biology Graduate Program, University of Nevada, Reno, NV, USA; ^2^ Department of Psychology, University of Western Ontario, London, Canada; ^3^ Department of Ecology, Evolution, and Organismal Biology, Kennesaw State University, Kennesaw, GA, USA

**Keywords:** long-term study, climate, environmental cues, phenology, reproductive performance

## Abstract

Animals use climate-related environmental cues to fine-tune breeding timing and investment to match peak food availability. In birds, spring temperature is a commonly documented cue used to initiate breeding, but with global climate change, organisms are experiencing both directional changes in ambient temperatures and extreme year-to-year precipitation fluctuations. Montane environments exhibit complex climate patterns where temperatures and precipitation change along elevational gradients, and where exacerbated annual variation in precipitation has resulted in extreme swings between heavy snow and drought. We used 10 years of data to investigate how annual variation in climatic conditions is associated with differences in breeding phenology and reproductive performance in resident mountain chickadees (*Poecile gambeli*) at two elevations in the northern Sierra Nevada mountains, USA. Variation in spring temperature was not associated with differences in breeding phenology across elevations in our system. Greater snow accumulation was associated with later breeding initiation at high, but not low, elevation. Brood size was reduced under drought, but only at low elevation. Our data suggest complex relationships between climate and avian reproduction and point to autumn climate as important for reproductive performance, likely via its effect on phenology and abundance of invertebrates.

## 1. Introduction

Environmental conditions such as climate can influence animal reproduction at different stages of the breeding cycle, including modulating breeding timing, investment and offspring survival [1,2]. Many animals use photoperiod as a general cue to determine when in the annual cycle to breed [3–5] and then rely on climate-based environmental cues to fine-tune their reproductive phenology [6,7]. This enables organisms to match favourable conditions such as seasonal peaks in food abundance that are necessary for raising young. For birds, spring temperature is the most common environmental cue associated with fine-tuning of breeding phenology [8,9], probably in part because spring temperatures affect the availability of invertebrates, a critical food source during reproduction [4,10,11]. The relationship between spring temperatures and the timing of avian breeding has received much attention in light of global climate change, which is causing spring temperatures to increase in many regions, leading many species to respond by breeding earlier [1,12–23]. However, temperature may not be the most reliable predictor of breeding phenology in all ecosystems or for all species, as the effects of climate change vary widely across environments and are not limited to changes in temperature [24]. For example, in the tropics, where precipitation may be a more important driver of insect abundance than temperature, rainfall is often the primary environmental cue associated with the start of breeding [25–27]. Understanding how animals in different environments respond to variation in different climatic conditions is critical to predicting how animal populations may respond to the many facets of global climate change.

In addition to the timing of reproduction, climatic conditions can influence reproductive investment and performance. Animals also use environmental cues to alter their reproductive investment to better match prevailing environmental conditions [28–30]. For example, several songbirds of an arid South African shrubland, including the spike-heeled lark (*Chersomanes albofasciata*), increased their clutch sizes when greater rainfall preceded the breeding season, presumably in anticipation of increased food availability following increased rainfall [30]. When animals fail to adjust their reproductive timing or investment to best match future conditions, reproductive performance may decrease. Food availability during the breeding season influences offspring survival [31,32] and the probability of survival may decrease if breeding timing and investment do not match peak food availability (referred to as a phenological mismatch) [10,33–36]. In songbirds, invertebrate prey abundance during the breeding season is a primary driver of variation in reproductive performance [11,33,37–42]. Additionally, abiotic conditions, such as ambient temperature, may also directly affect offspring survival [43]. Therefore, climatic conditions can have both direct and indirect effects on bird reproductive performance, making studying these relationships even more pressing in the face of global climate change.

Previous correlational studies investigating the relationships between breeding phenology, reproductive performance, and environmental variation have taken place primarily at elevations near sea level [11–15,17,18,23]. However, high-elevation temperate ecosystems also experience warming and extreme annual swings in environmental conditions, including variation in snowfall [44–51]. Climatic variables may respond differently to climate change or have different ecological effects at sea level versus in montane environments, so findings from previous studies at sea level may not be generalizable to all environments. Even within montane environments, animals in the same population may experience vastly different conditions that can affect their breeding phenology and reproductive performance because climatic conditions often vary across elevations. For example, in semi-arid regions, accumulated snowfall at higher elevations provides water to lower elevations prone to drought throughout the year [52]. Thus, high snow years can alleviate drought conditions at surrounding lower elevations, while low snow years can exacerbate them and lead to negative effects on invertebrate food availability [53–55], possibly influencing avian reproductive performance. Given these complex relationships, spring temperatures may not be the only variable driving breeding phenology and reproductive performance within these systems. In fact, previous work conducted in a montane setting showed that snow depth was a better predictor of breeding timing than spring temperatures [56], possibly because snowpack can delay invertebrate emergence [57]. Furthermore, snow has the unique potential to directly affect reproductive phenology by physically preventing access to nest sites or materials until snowmelt, especially in cavity- or ground-nesting species. Because the influence of climatic factors on breeding phenology and reproductive performance can vary at such small spatial scales in montane environments, species in these ecosystems may not fit the generalizations of previous work, but it is critical to understand how such species may cope with long-term climate trends.

In this study, we explored how annual variation in climate over 10 years (2013–2022) was associated with the breeding phenology and reproductive performance of a resident mountain chickadee population (*Poecile gambeli)* at low elevation (range: 1965 m–2070 m) and high elevation (range: 2380 m–2590 m) in the northern Sierra Nevada. Mountain chickadees are highly resident cavity-nesting songbirds that feed on invertebrates during the breeding season, which occurs from April to August [58,59]. Previously, with a shorter-term dataset of 5 years, we showed that birds living at low and high elevations responded differently to climate extremes: low elevation birds exhibited the lowest reproductive output (brood size) during low water years, while high elevation birds had the lowest brood size during heavy snow years [60]. However, given the relatively short period of that study, we were limited in our ability to statistically assess the contributions of temperature and other climate variables to reproductive phenology and success.

**Figure 1.**
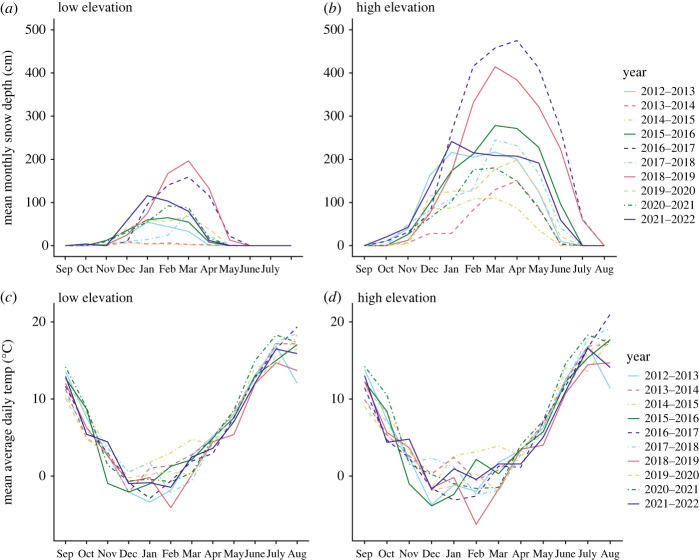
Variation in mean monthly snow depth (*a,b*), and mean monthly average daily temperatures (°C) (*c,d*), at two elevational sites at Sagehen Experimental Forest, CA, USA.

**Figure 2.**
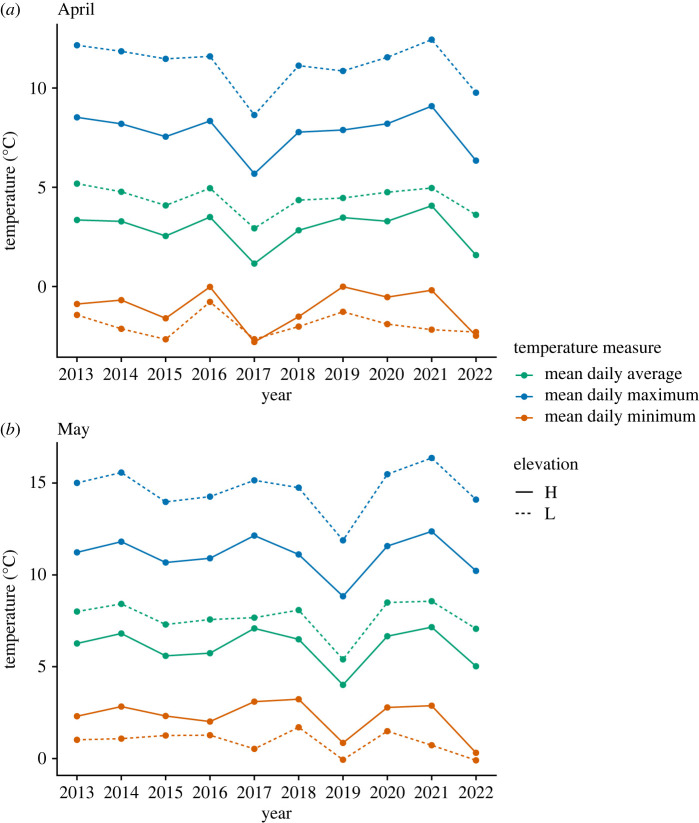
Mean average, minimum and maximum daily temperatures (°C) in (*a*) April and (*b*) May at two elevational sites at Sagehen Experimental Forest, CA, USA from 2013 to 2022.

Birds at our study site were exposed to ample annual variation in temperatures and snow depth over the last 10 years (figures 1 and 2; electronic supplementary material, figures S1 and S2), providing the unique opportunity to study how variation in climatic conditions may be associated with breeding phenology and reproductive performance. We used sliding window analyses [61] to identify the best climatic predictors of mountain chickadee breeding phenology and breeding performance separately for low and high elevations. We hypothesized that chickadees adjust their reproductive timing and investment based on climatic conditions (such as temperature) that have been reported to affect phenology of invertebrates—their primary food source [58,59]. Furthermore, we hypothesized that heavy and longer-lasting snow cover would decrease access to nests sites and nesting material and delay invertebrate emergence, thereby resulting in delayed first egg dates [57]. We hypothesized that if birds were not able to shift their breeding phenology to match invertebrate phenology, or if climatic conditions led to a lower overall abundance of invertebrates, they would have lower reproductive performance. Based on the hypotheses above, we predicted that: (i) warmer spring temperatures will lead to advances in breeding phenology (earlier first egg dates) at both high and low elevations; (ii) first egg dates will be more influenced by snow depth than temperature at high elevation but not at low elevation; (iii) less precipitation will lead to lower reproductive investment at (drought-prone) low elevation, but not at high elevation; and (iv) less precipitation will lead to lower reproductive performance (e.g. smaller broods, lower nestling mass) at low elevation, but not at high elevation.

## 2. Methods

### 2.1. Climate data

We obtained weather data from three Snow Telemetry (SNOTEL) stations located in close proximity to our field site, which are maintained by the United States Department of Agriculture Natural Resources Conservation Service (https://wcc.sc.egov.usda.gov/nwcc/): Independence Camp (SNOTEL site 539, elevation 2128 m, coordinates: 39.45, −120.29), Independence Creek (SNOTEL site 540, elevation 1962 m, coordinates: 39.49, −120.28; combined, these stations represent the range of elevations in our low elevation study area, 1965 m-2070 m) and Independence Lake (SNOTEL site 541, elevation 2541 m, coordinates: 39.43, −120.31; this station is directly in the middle of our high elevation study area). These were the only SNOTEL stations in or near our study area, and these stations collect hourly and daily weather data including precipitation, air temperature, soil moisture and soil temperature. We used the following daily climate variables in our analyses: precipitation accumulation (mm), snow depth (cm), snow water equivalent (mm), average, maximum, and minimum air temperature (°C), soil moisture (%) taken at a depth of 5 cm (2 in) and 51 cm (20 in), and soil temperature (°C) taken at a depth of 5 cm, 20 cm (8 in) and 51 cm. We recalculated precipitation accumulation to start on 1 September of each year to coincide with the end of the breeding season. We averaged sites 539 (elevation 2128 m) and 540 (elevation 1962 m), which are 4.5 km and 6 km away from our low elevation site, respectively, to correctly capture the climatic conditions across the elevation range at our low elevation study area.

### 2.2. Breeding data

We monitored breeding behaviour over 10 years (2013–2022) at the Sagehen Experimental Forest (Sagehen Creek Field Station, University of California Berkeley), CA, USA. During the breeding season (April–August), we maintained approximately 350 nest-boxes at two elevational sites separated by approximately 3.49 km: ‘low' (range: 1965 m–2070 m; coordinates: 39.44350, −120.243248) and ‘high' (range: 2380 m–2590 m; coordinates: 39.42402, −120.315015) [60]. The vegetation at both elevations is comprised of mostly coniferous trees, with Jeffrey pine (*Pinus jeffreyi*), sugar pine (*Pinus lambertiana*), lodgepole pine (*Pinus contorta)* and white fir (*Abies concolor*) being the most common at low elevation, and western white pine (*Pinus monticola*), lodgepole pine, mountain hemlock (*Tsuga mertensiana*), and red fir (*Abies magnifica*) being the most common at high elevation. We started visiting nest-boxes weekly in mid-April to detect evidence of nest building. Once nest building was detected, we checked nest-boxes one to two times per week until we detected egg laying (in all cases, we detected egg laying before incubation started), allowing us to calculate the first egg date because chickadees lay one egg per day [60]. Our timing never resulted in missed nests and all nests were detected during the nest building stage (egg laying never occurred before 3 May, which is the earliest recorded first egg date at low elevation). Once eggs were detected, we started checking nests every 2–3 days until we detected incubation and used the differences in the number of eggs prior and the number of eggs when incubation was detected to estimate start of incubation (chickadees start incubating in the morning after the last egg was laid). Once we detected incubation, we next checked the nests 12 days later (incubation takes 13–14 days), and if hatching was not detected, we kept checking the nests for hatching every day until we detected hatching. Once the hatching date was recorded, we banded and measured all nestlings on day 16. These methods allowed precise measurements of all phenological events, and we have never missed a nest in our nest-boxes at any of the breeding stages. Clutch size was recorded as the number of eggs in the nest at the beginning of incubation. Fledgling body mass and brood size were recorded on day 16 after hatching when all nestlings were weighed and banded with a unique numeric aluminium band issued by the United States Geological Survey Bird Banding Laboratory. Fledging time varies between day 20 and 24 after hatching. We evaluated within-nest variation in nestling condition by calculating the coefficient of variation in nestling mass (CV; (s.d÷mean)×100). We used the following breeding parameters in our analyses: first egg date, clutch size, brood size, mean nestling mass and coefficient of within-nest variation in nestling mass. Our dataset included a total of 998 nests: 413 nests at high elevation and 585 nests at low elevation (electronic supplementary material, table S4). We only used initial breeding attempts for each season, i.e. renesting or second nesting attempts from the same season were removed from the dataset prior to analyses. Mountain chickadees typically raise only one brood per season [59]; second broods and renests were quite unusual during our study period (only 13 second broods and 25 renests were recorded over 10 years).

### 2.3. Climatic predictors of variation in breeding timing and reproductive performance

We conducted sliding window analyses to test which climate variables best-predicted variation in timing of breeding and reproductive performance across years [13,18,27,61–63]. We ran separate sliding window analyses for each combination of climate and breeding variables to determine the climatic periods associated with variation in each breeding variable [61]. We used the ‘slidingwin' function from the ‘climwin' package in R v. 4.1.3 [64] and searched for absolute windows in all analyses (windows in which variation in the climate variable during that period best-explained variation in a breeding response variable across years) [65]. We used the mean of the climate values within each window as our explanatory variable for all climate variables. We required that the climate windows be at least 14 days long, as smaller windows are probably biologically irrelevant [65] and searched for windows that began as early as 1 September of the year preceding a given breeding season. For first egg date, we searched for windows up to the earliest recorded first egg date at each elevation. We searched for windows up to the earliest first egg date plus 9 days for clutch size and plus 40 days for all other breeding parameters (brood size, mean nestling mass and coefficient of within-nest variation in nestling mass). By using 40 days past the earliest first egg date, we account for egg laying (9 days maximum), incubation and hatching (15 days) and days in the nest (16 days). We compared the predictive power of climate windows from the same climate covariates using the Akaike information criterion adjusted for small sample sizes (AICc). We compared each model with a different climate window to a baseline null model that did not include climate covariates to measure the improvement of the climate model over the null (ΔAICc). All windows within 2 ΔAICc of the top climate model were considered equivalent in performance. If the windows performing equally overlapped substantially in time (for example, a window from 14 October to 4 December and a window from 14 October to 5 December), we chose the climate window with the lowest ΔAICc to include in a final model for each breeding variable. We conducted these analyses separately for low and high elevations because we expected that the responses of breeding phenology (first egg date) and reproductive performance (clutch, brood, mean nestling mass and coefficient of within-nest variation in nestling mass) would differ between elevations based on our previous work [60].

We modelled the effect of climate on clutch size and brood size using the generalized Poisson distribution from the glmmTMB package [66], and modelled first egg date, nestling mass, and coefficient of within-nest variation in nestling mass using a Gaussian distribution. Coefficient of within-nest variation in nestling mass was log-transformed to improve residual fit. We used the ‘DHARMa' package in R to simulate residuals, check residual fit, and check for model misspecification problems [67].

Because sliding window analyses compare a large number of models, the chances of obtaining a false positive are high [65]. Therefore, we tested whether our top windows performed better than what would be expected by chance by comparing the improvement of our top model for each climate variable over the null model (ΔAICc) to the ΔAICc of 100 randomizations for each breeding variable [65]. We randomized our observed data by reassigning data from a single year to the same random year, but the climate data were kept matching their original year [27]. This randomization process keeps sets of yearly breeding values together rather than randomly mixing breeding values across years, which would eliminate the variation between years that is inherent to this type of data. We compared the ΔAICc of the top window in each of the 100 randomizations with the ΔAICc of the top window from the original dataset. Because we were specifically interested in testing whether the observed relationship was different from what can be expected by chance, we only compared our observed models to randomizations with parameter estimates that matched the sign of the original model [27]. If five or fewer of the top windows from these randomizations performed better (had a more negative ΔAICc) than the top model from the original dataset, we concluded that the observed top model was probably not a false positive (electronic supplementary material, tables S5–S9, referred to as P-rand). Climate windows that were likely false positives were not considered in any further analyses.

### 2.4. Final climate models

After obtaining the top windows for each climate variable from the sliding window analyses, we created final models for each breeding parameter at each elevation. First, we constructed single-variable models for each top climate window-breeding parameter combination and excluded variables from the final models that were not significant predictors of breeding timing or reproductive performance based on likelihood ratio tests. Next, we compared model performance of similar climate variables with overlapping top windows. Since climate variables such as average, maximum and minimum daily air temperature probably have similar effects, when windows were similar across these variables, we compared AICc values between single-variable models and we present the variable from the model with the lowest AICc. We checked for collinearity among the remaining covariates using Pearson's correlation coefficient. For correlated variables, we compared the performance (AICc) of univariate models to select the best predictor. Finally, model selection was performed in which combinations of the remaining uncorrelated variables were compared using AICc. We report all competing top models within 2 AICc values of the model with the lowest AICc score.

## 3. Results

### 3.1. Climate patterns

Our long-term field site in the Sagehen Experimental Forest (Sagehen Creek Field Station, University of California Berkeley) has experienced large annual climatic swings that have resulted in unpredictably alternating years of deep snow and low precipitation over the last 10 years (figure 1). Snow depth has been consistently higher across years at high elevation compared to low elevation, and high elevation has experienced greater interannual variation in snow depth (figure 1). Both elevations at our field site have experienced warming trends over the past 27 years, with the largest changes seen in increasing minimum daily temperatures in July–August (electronic supplementary material, tables S1–S3; figures S3 and S4). Spring temperatures, important phenological cues in many taxa [2,10,11], also show significant warming trends during the same period at both elevations in April and at high elevation in May. During the 10 years in which we conducted our study, although there was ample variation across years (figure 2), there were no statistically significant trends in spring temperature (electronic supplementary material, figures S3 and S4), and the only significant warming trends were in August (electronic supplementary material, tables S1–S3).

### 3.2. Variation in breeding parameters across years by elevation

**Figure 3.**
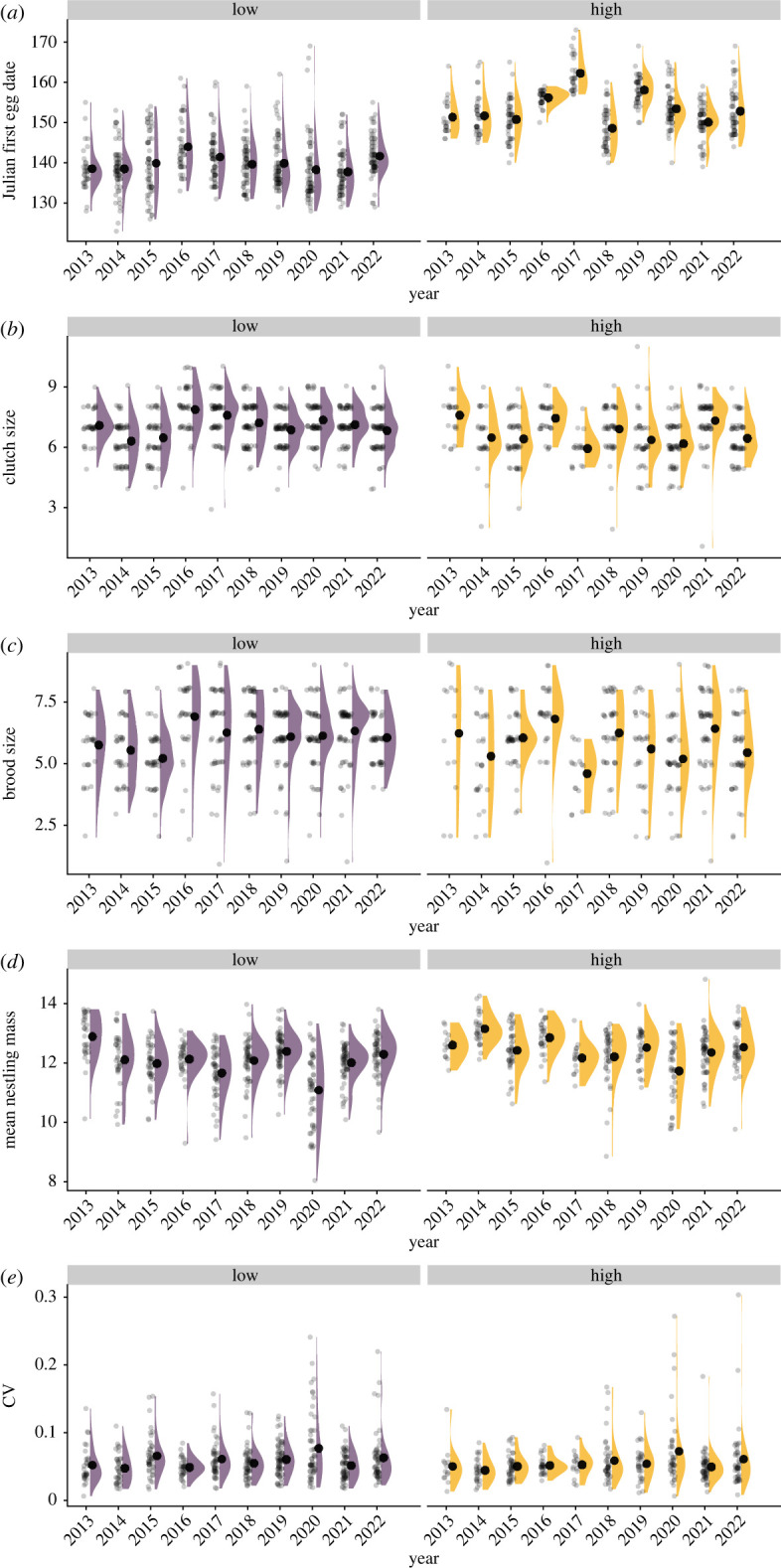
Variation in breeding parameters across years for mountain chickadees at low and high elevations at Sagehen Experimental Forest, CA, USA: (*a*) first egg date; (*b*) clutch size; (*c*) brood size; (*d*) mean nestling mass; (*e*) coefficient of within-nest variation in nestling mass (CV). The mean values are shown as large black dots, the raw data are shown as small black dots and the distributions of the raw values are shown in purple for low elevation and orange for high elevation.

During our 10-year study, there was considerable variation across years in mountain chickadee breeding initiation (first egg dates) at both low (*χ*^2^ = 41.36, *p* < 0.001) and high (*χ*^2^ = 204.04, *p* < 0.001) elevation sites, but first egg date did not increase or decrease consistently during the 10 years of this study (low: *t* = 0.06, *p* = 0.95; high: *t* = −0.15, *p* = 0.87) (figure 3*a*). There was significant across-year variation in clutch size (low: *χ*^2^ = 102.59, *p* < 0.001; high: *χ*^2^ = 59.94, *p* < 0.001), brood size (low: *χ*^2^ = 55.77, *p* < 0.001; high: *χ*^2^ = 48.07, *p* < 0.001), and mean nestling mass (low: *χ*^2^ = 117.27, *p* < 0.001; high: *χ*^2^ = 67.36, *p* < 0.001; figure 3*b–d*). Coefficient of within-nest variation in nestling mass varied significantly across years at low (*χ*^2^ = 29.19, *p* < 0.001) but not at high elevation (*χ*^2^ = 12.04, *p* = 0.211) (figure 3*e*).

### 3.3. Low elevation: climatic variation, breeding phenology and reproductive performance

**Figure 4.**
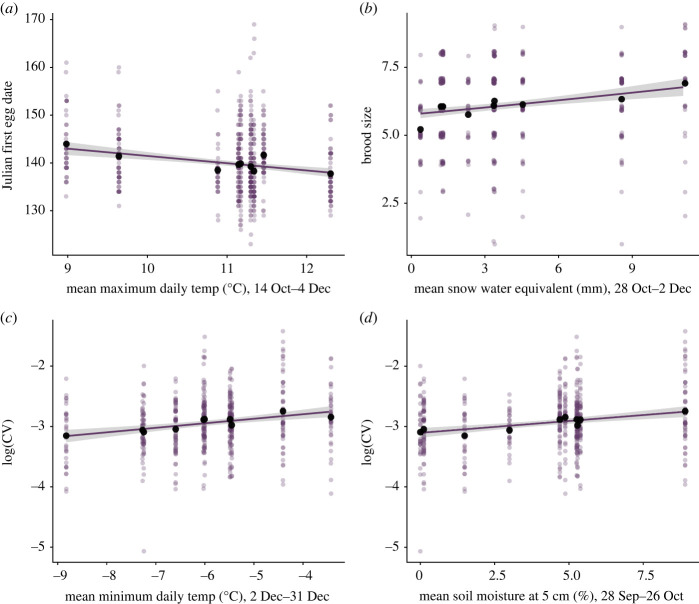
Climatic predictors of variation in breeding parameters at low elevation. Relationships between: (*a*) maximum daily temperatures (°C) in autumn and first egg date; (*b*) snow water equivalent (mm) in autumn and brood size; (*c*) minimum daily temperatures in December (°C) and log-transformed coefficient of within-nest variation in nestling mass (CV); and (*d*) mean soil moisture measured at 5 cm depth (%) from late September to late October and log-transformed CV. Raw data are displayed as purple points, means are displayed as black points and model fit line with 95% confidence intervals are included.

Maximum daily temperature from 14 October to 4 December was the only significant climate predictor of first egg date at low elevation, with higher temperatures in the autumn associated with earlier egg laying in the following breeding season (table 1*a*; electronic supplementary material, table S5; figure 4*a*; electronic supplementary material, figure S5). There were no significant climatic predictors of clutch size at low elevation (electronic supplementary material, table S6), but higher snow water equivalent from 28 October to 2 December was associated with larger brood size (electronic supplementary material, table S7; figures 1*b* and 4*b*; electronic supplementary material, figure S8). There were no significant climatic predictors of mean nestling mass at low elevation (electronic supplementary material, table S8), but higher minimum daily temperatures from 2 to 31 December and higher soil moisture from 28 September to 26 October were associated with greater variation in nestling mass within nests (coefficient of variation) at low elevation (electronic supplementary material, table S9; figures 1*c* and 4*c*,*d*; electronic supplementary material, figure S10).

**Table 1.** Climatic predictors of breeding phenology, investment and reproductive performance at low elevation.

(*a*) linear model results for low elevation first egg date top model				
fixed effects	parameter estimate	s.e.	*t*-statistic	*p*-value
intercept	156.69	3.43	45.65	<2 × 10^−16^***
mean maximum daily temp (°C) (14 Oct–4 Dec)	−1.52	0.31	−4.91	1.19 × 10^−16^***
(*b*) linear model results for low elevation brood size top model				
fixed effects	parameter estimate	s.e.	*z*-statistic	*p*-value
intercept	1.75	0.01	112.02	<2 × 10^−16^***
mean snow water equivalent (mm) (28 Oct–2 Dec)	0.01	0.01	4.76	1.98 × 10^−6^***
(*c*) linear model results for low elevation CV top models				
model 1				
fixed effects	parameter estimate	s.e.	*t*-statistic	*p*-value
intercept	−2.49	0.09	−25.25	<2 × 10^−16^***
mean minimum daily temp (°C) (2 Dec–31 Dec)	0.07	0.02	4.59	5.54 × 10^−6^***
AICc	590.35			
model 2				
fixed effects	parameter	s.e.	*t*-statistic	*p*-value
intercept	−3.10	0.04	−76.25	<2 × 10^−16^***
mean soil moisture at 5 cm (%) (28 Sep–26 Oct)	0.04	0.01	4.74	2.84 × 10^−6^***
AICc	589.06			

### 3.4. High elevation: climatic variation, breeding phenology and reproductive performance

**Figure 5.**
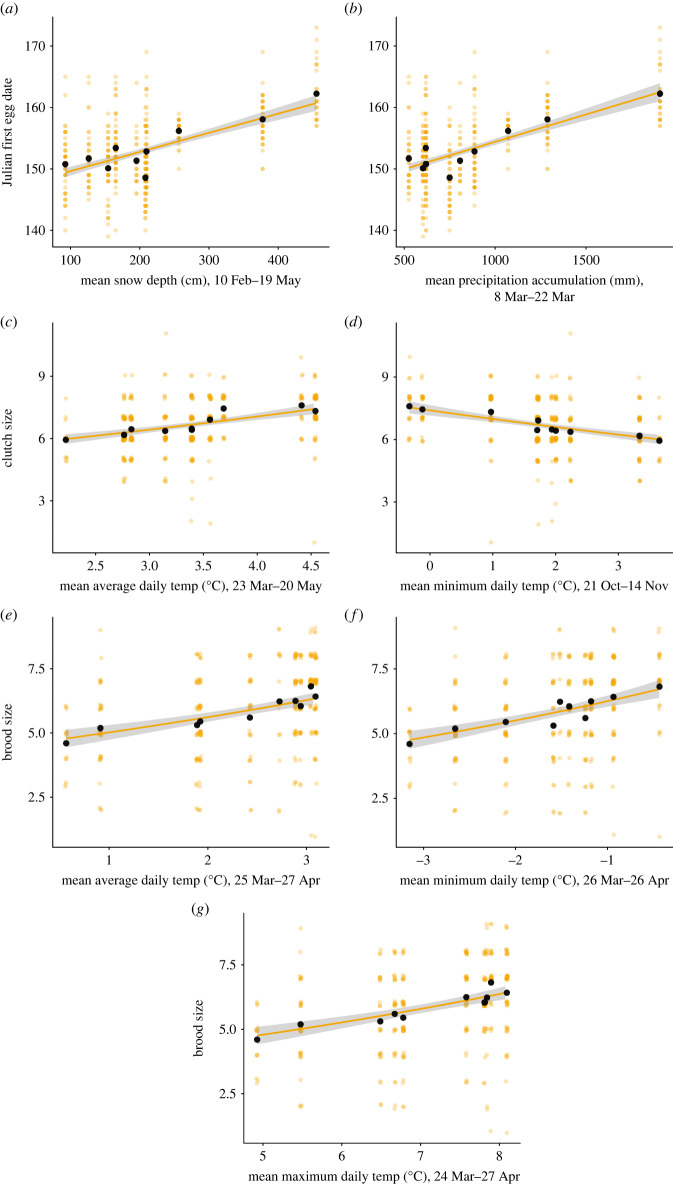
Climate predictors of variation in breeding parameters at high elevation. Relationships between: (*a*) snow depth from early February to mid-May and first egg date; (*b*) precipitation accumulation (mm) in March and first egg date; (*c*) average daily temperatures (°C) from late March to late May and clutch size; (*d*) minimum daily temperatures (°C) from late October to mid-November and clutch size; (*e*) average daily temperatures (°C) from late March to late April and brood size; (*f*) minimum daily temperatures (°C) from late March to late April and brood size; and (*g*) maximum daily temperatures (°C) from late March to late April and brood size. Raw data are displayed as orange points, means are displayed as black points and model fit lines with 95% confidence intervals are included.

First egg date at high elevation was significantly associated with precipitation accumulation by March: egg laying occurred later in the years with more snow by 8–22 March (electronic supplementary material, table S5; table 2*a*, figure 5*b*; electronic supplementary material, figure S6). Higher snow depth in spring was also significantly associated with later egg laying (figure 5*a*), but precipitation accumulation explained more variation in first egg dates across years (based on AICc comparison).

**Table 2.** Climatic predictors of breeding phenology, investment and reproductive performance at high elevation.

(*a*) linear model results for high elevation first egg date top model				
fixed effects	parameter estimate	s.e.	*t*-statistic	*p*-value
intercept	1.45 × 10^2^	0.61	237.28	<2 × 10^−16^***
mean precipitation accumulation (mm) (8 Mar–22 Mar)	8.90 × 10^−3^	6.61 × 10^−4^	13.47	<2 × 10^−16^**
(*b*) linear model results for high elevation clutch size top models				
model 1				
fixed effects	parameter estimate	s.e.	*z*-statistic	*p*-value
intercept	1.57	0.04	32.47	<2 × 10^−16^***
mean average daily temp (°C) (23 Mar–20 May)	0.09	0.01	6.80	1.01 × 10^−11^***
AICc	1208.9			
model 2				
fixed effects	parameter estimate	s.e.	*z*-statistic	*p*-value
intercept	2.00	0.02	116.99	<2 × 10^−16^***
mean minimum daily temp (°C) (21 Oct–14 Nov)	−0.057	0.008	−6.82	9.23 × 10^−12^***
AICc	1208.9			
(*c*) linear model results for high elevation brood size top models				
model 1				
fixed effects	parameter estimate	s.e.	*z*-statistic	*p*-value
intercept	1.50	0.04	33.11	<2 × 10^−16^***
mean average daily temp (°C) (25 Mar–27 Apr)	0.11	0.02	6.23	4.54 × 10^−10^***
AICc	1212.0			
model 2				
fixed effects	parameter estimate	s.e.	*z*-statistic	*p*-value
intercept	1.09	0.11	10.08	<2 × 10^−16^***
mean maximum daily temp (°C) (24 Mar–27 Apr)	0.09494	0.01501	6.325	2.54 × 10^−10^***
AICc	1210.9			
model 3				
fixed effects	parameter estimate	s.e.	*z*-statistic	*p*-value
intercept	1.96	0.03	58.26	<2 × 10^−16^***
mean minimum daily temp (°C) (26 Mar–26 Apr)	0.12	0.02	6.23	4.57 × 10^−10^***
AICc	1211.9			

Higher average daily temperatures from 23 March to 20 May and lower minimum daily temperatures from 21 October to 14 November were significantly associated with larger clutches (electronic supplementary material, table S6; figures 2*b* and 5*c*,*d*; electronic supplementary material, figure S7) while higher mean, maximum and minimum daily temperatures in spring (late March to late April) were associated with larger brood sizes (electronic supplementary material, table S7; table 2*c*, figure 5*e–g*; electronic supplementary material, figure S9). Notably, for brood size, average, maximum and minimum daily air temperatures all had the same top window from the sliding window analysis. No measured climatic predictors were significantly associated with variation in mean nestling mass or coefficient of within-nest variation in nestling mass within nests (electronic supplementary material, tables S8 and S9).

## 4. Discussion

Our most important finding was that annual variation in spring temperature was not significantly associated with initiation of breeding (first egg date) in mountain chickadees at either elevation. These results clearly contrast with our first prediction and the results of similar phenological studies, although most previous studies have been conducted at elevations closer to sea level [11–15,17,18,23]. Instead, in our study, autumn temperatures and snow depth were associated with initiation of breeding. In support of our second prediction, snow depth was a significant predictor of breeding timing at high but not at low elevation. In contrast to our third prediction, clutch size was not associated with any climatic conditions at low elevation; rather, clutch size increased with increasing spring temperatures and decreased with increasing autumn temperatures at high elevation. Low, but not high elevation birds experienced decreased brood size in years with lower snow water equivalent, which is usually associated with drought, supporting our fourth prediction. Importantly, climatic variation was also associated with variation in reproductive performance metrics at high and low elevations, but different climate variables were important at different elevations. Our results linking autumn climate with reproductive timing and performance suggest that autumn climate may directly affect invertebrate abundance the following spring and chickadees may use cues related to invertebrates to time their breeding. Overall, our results emphasize that the environmental cues associated with breeding phenology and reproductive investment in birds may not be generalizable across all environments and that variation in invertebrate emergence and abundance may be associated with climatic conditions other than spring temperature.

### 4.1. Spring climate, reproductive phenology and performance

#### 4.1.1. Reproductive phenology

Spring temperatures were not associated with first egg dates at either elevation, suggesting that chickadees in the northern Sierra Nevada probably relied on other environmental cues to time initiation of breeding.

At high elevation, annual variation in snow depth was significantly associated with differences in breeding timing, with chickadees initiating egg laying later in years with more snow. Notably, precipitation accumulation from September to March is the top explanatory variable for breeding timing at high elevation, where greater precipitation accumulation (in the form of snow) is associated with later egg laying. At low elevation, such association was not present, probably owing to the overall lower snow depth at this location (figure 1). The association between snow accumulation and reproductive timing at high elevation can probably be explained by three mechanisms: (i) high snow depth prevents access to some nesting cavities, and since mountain chickadees are secondary cavity nesters [59], they cannot compensate by excavating their own cavity; (ii) high snow depth buries nest building materials [59]; and (iii) high snow depth may delay invertebrate emergence and peak abundance. Although there is limited research on the influence of snow on invertebrate phenology in montane environments, a study conducted over 10 years in the Arctic showed that snow melt out date was a better predictor of arthropod emergence than spring temperature in six of the nine most abundant taxa collected [68]. At 2400 m in altitude, our high-elevation site experiences harsh winter conditions including heavy snow that may last well into July (figure 1), which may influence invertebrate phenology in a similar way to more northern latitude locations.

While most studies on bird reproductive phenology link spring temperature to changes in egg-laying dates, a few studies do show that snow influences phenology at high elevations [69–71]. Our result is also consistent with another study on the closely related coal tit (*Periparus ater*), which showed that at higher elevations, reproductive timing was limited by snow melt out date, which was a better predictor of phenology than spring temperature [56].

However, other studies on the breeding phenology of mountain chickadees from central British Columbia, Canada [19,72] and southern California, USA [73] showed that spring temperature was the primary climate variable that predicted breeding timing. Notably, these studies are from other regions that experience different climatic conditions and variation: one study was from a southern part of the Sierra Nevada [73] and the other two studies were from Canada [19,72]. Additionally, Coe *et al*. used a predetermined 7-day window of time for spring temperatures rather than testing multiple windows of time, and the study only spanned 5 years. Such small windows based on *a priori* assumptions may be less biologically relevant and the shorter span of the study may also not be sufficient to detect long-term patterns [61,65,74]. In fact, in a recent review using data from long-term ecological research sites, Cusser *et al*. [75] demonstrated that in most cases, 10 or more years of data are needed to obtain repeatable results of complex ecological associations. Additionally, in the aforementioned chickadee studies, temperature and snow measurements did not come from the exact study locations, but from rather distant locations and at lower elevations [19,72,73]. It is possible that these less precise measurements do not reflect fine-scale differences in climate variables, especially in the mountains, where elevation-specific differences appear to be critical to explaining variation in breeding phenology and reproductive performance [76]. Our study used weather stations located at precise and relevant elevations, allowing accurate measurements of environmental conditions directly at our sites. Nonetheless, it is likely that conditions specific to the geographical location of a population, even of the same species, may uniquely shape phenological responses.

#### 4.1.2. Reproductive performance

*Clutch and brood size*. We identified no climatic predictors of clutch size at low elevation, but warmer spring temperatures were associated with larger clutches at high elevation. This finding may suggest that high elevation birds adjust their reproductive investment in response to spring temperature. Warmer spring temperatures contribute to faster snow melting, which in turn can lead to earlier emergence of invertebrates and better conditions for raising young [68]. High elevation birds have a much smaller window of time to breed compared to low elevation birds because they are constrained by heavy snow in the spring and must finish breeding early enough to moult and begin caching food to survive winter (mountain chickadees are a food-caching species) [59]. If insect phenology is earlier in warmer springs, high elevation birds may be better able to match peak food conditions, raise more offspring, and finish breeding at an optimal time. Thus, laying larger clutches may pay off following warmer springs. Notably, other studies that found an association between clutch size and temperature show that this relationship was mediated by first egg date, where warmer spring temperatures led to earlier laying which was associated with larger clutches [16,77–79]. By contrast, our results suggest that birds at high elevation are shifting their reproductive investment with temperature, but unrelated to first egg date.

At high elevation, brood size was positively associated with spring temperatures. Since both clutch and brood sizes were associated with spring temperatures at high elevation, this suggests that high elevation birds may have shifted their reproductive investment (clutch size) to match favourable conditions (invertebrate abundance), thus increasing reproductive output (brood size).

### 4.2. Autumn climate, reproductive phenology and performance

#### 4.2.1. Reproductive phenology

At low elevation, warmer temperatures from October to December before reproduction were associated with earlier egg laying. It is not clear why this relationship was present, but it is unlikely that chickadees use autumn temperatures as a cue for reproduction occurring at least five months later. Instead, it is more likely that autumn temperatures may affect the phenology and abundance of invertebrates in the following spring [80], which in turn could directly affect the initiation of breeding each year [81]. It is well known that some birds use the phenology of other organisms as cues in addition to climatic cues to time reproductive events. For example, some species use plant phenology (green-up, budburst) as a cue, particularly in deciduous forests [82,83]. However, because our study took place in coniferous forest, with no clear green-up or budburst event, it is unlikely that our birds are using plant phenology as a cue. Although it remains relatively unstudied, birds may also use the emergence of certain invertebrate groups as a cue [81]. Therefore, it is likely that insect phenology, influenced by previous autumn temperatures [80], may best explain our observed relationship between autumn temperatures and the initiation of breeding in chickadees.

#### 4.2.2. Reproductive performance

*Clutch and brood size*. While we found a negative association between clutch size and autumn temperatures at high elevation, it is unlikely that birds are using autumn temperatures as a direct cue to adjust their clutch sizes. Instead, increasing autumn temperatures can delay the initiation of invertebrate dormancy or result in increased energy expenditure during dormancy, potentially resulting in higher invertebrate mortality [80,84]. Furthermore, repeated freeze–thaw events, which are more frequent with higher autumn and winter temperatures, can have negative impacts on insect abundance [84]. Thus, warmer autumn and winter temperatures may create suboptimal breeding conditions the following year owing to low food availability. Birds may adjust clutch sizes in anticipation of such suboptimal conditions by using some indicators of future invertebrate timing and abundance that are directly proportional to autumn temperatures.

At low elevation, brood size was positively associated with autumn and early winter snow water equivalent (late October to early December), suggesting that more accumulated moisture early in the winter season increases reproductive performance at low elevation. Because lower elevation montane regions rely on snowpack from higher elevations for their water supply throughout the reproductive season [52], when sufficient snowpack is not available, low water conditions (e.g. drought) may negatively affect invertebrate abundance at low montane elevations [53–55]. Notably, brood size was not associated with snow at high elevation. Snow may have been an important driver of reproductive output at low but not at high elevation because high elevation birds may not experience the same negative effects on invertebrate abundance during low snow years as low elevation birds. This is likely because high elevation receives much more snow than low elevation, so high elevation may have sufficient snowmelt to sustain invertebrates during low snow years.

*Fledgling condition*. Mean nestling mass was not associated with any climatic variables at either elevation, but minimum daily temperatures in December and soil moisture from late September to late October were positively associated with within-nest variation in nestling mass at low elevation. Nestling mass is an indicator of nestling condition, where larger nestlings are more likely to survive and be recruited into the population [85–90]. Therefore, our results suggest that warmer early winter temperatures and increased soil moisture in the autumn may have negative effects on nestling fitness because parents cannot adequately feed all nestlings, resulting in high variation in condition within nests.

Warmer winters and increased soil moisture in the autumn are probably associated with a decreased availability of invertebrate prey the following breeding season for two reasons. First, the abundance of invertebrates is probably related to water availability in spring and summer [53–55], and warmer temperatures decrease the amount of snow that accumulates during the winter season to provide water during the drier months [45,47,91]. Increased soil moisture in autumn indicates that autumn precipitation is falling in the form of rain or that snow is melting and penetrating the topsoil layer; in both cases, snow is not stored, which may create drought conditions for the following summer. Second, survival of invertebrates overwintering underground may be affected by the depth of soil freezing during the coldest times of the year [92]. Winter snowpack insulates the soil and can prevent deep soil freezing. If snow melts immediately owing to warm temperatures, less snow will accumulate over the winter, and the lack of insulating snowpack will cause the soil to freeze to a greater depth later in the winter season [92]. Greater soil freezing depth can have detrimental effects on the survival of invertebrates overwintering underground. For example, a long-term study in New Hampshire showed that beetle abundance the following year decreased with increasing winter temperatures, which the authors attribute to the associated reduction in snow cover that protects overwintering beetles from cold temperatures [93]. As generalist arthropodivores, mountain chickadees regularly feed their young invertebrates that winter underground, including beetles such as *Dichelonyx* sp. (Family: Scarabaeidae) [94]. Thus, it is possible that increased soil moisture in the autumn is associated with increased within-nest variation in nestling mass at low elevation because warmer temperatures in the autumn prevent snow accumulation, which may be critical to the overwinter survival of insect prey. However, this could not be the main explanation because there was no significant association between snow levels and within-nest variation in nestling mass at low elevation, suggesting that autumn temperatures and soil moisture may directly affect the emergence and abundance of invertebrates.

Notably, there were no climatic predictors of within-nest variation in nestling mass at high elevation, suggesting that high elevation birds do not experience the same negative effects from low snow years. This is likely because the snow cover is sufficient at high elevation, even during low snow years, to prevent deep soil freezing. Although earlier first egg dates at low elevation were associated with warmer autumn temperatures, higher within-nest variation in nestling mass was also associated with warmer autumn to early winter temperatures in December, suggesting that even if low elevation birds shift their breeding timing to be earlier when autumn temperatures are warmer, they may still struggle to feed nestlings.

## 5. Conclusion

Our results suggest that annual variation in spring temperature was not associated with differences in reproductive phenology in mountain chickadees in the northern Sierra Nevada, in contrast to many previous studies of avian species (see references above). Instead, among the variables we tested, annual variation in spring snow levels was associated with significant differences in reproduction phenology at high but not at low elevation. Variation in autumn and early winter climate, on the other hand, was associated with differences in reproductive phenology and performance, probably owing to the link between autumn and winter conditions and the phenology and abundance of invertebrates in spring and summer. Furthermore, different climatic variables appear to affect the breeding phenology and reproductive performance of chickadees at different elevations, highlighting the complex relationships between variation in climate and reproduction across an elevational gradient separated by only 3.49 km in distance and 500 m in altitude. Our study highlights the need to invest more effort in understanding the ecology of montane ecosystems and how the animals that live in these ecosystems respond to global climate change. Future research on food availability, including intensive long-term monitoring of invertebrates across elevations, will be critical to understanding the mechanisms driving variation in breeding timing and reproductive performance.

## Data Availability

Data and code underlying the results of this study are available on Dryad: https://doi.org/10.5061/dryad.ngf1vhj05 [95]. Supplementary material is available online [96].
